# Host–microbe interactions in the nasal cavity of dogs with chronic idiopathic rhinitis

**DOI:** 10.3389/fvets.2024.1385471

**Published:** 2024-08-12

**Authors:** Zhe Wang, Lyndah Chow, Sunetra Das, Renata Impastato, Alison C. Manchester, Steven Dow

**Affiliations:** Department of Clinical Sciences, College of Veterinary Medicine and Biomedical Sciences, Colorado State University, Fort Collins, CO, United States

**Keywords:** canine, microbiome, nose, immune, cilia, cytokine

## Abstract

Chronic rhinitis (CR) is a frustrating clinical syndrome in dogs and our understanding of the disease pathogenesis in is limited. Increasingly, host–microbe interactions are considered key drives of clinical disease in sites of persistent mucosal inflammation such as the nasal and oral cavities. Therefore, we applied next generation sequencing tools to interrogate abnormalities present in the nose of dogs with CR and compared immune and microbiome profiles to those of healthy dogs. Host nasal cell transcriptomes were evaluated by RNA sequencing, while microbial communities were assessed by 16S rRNA sequencing. Correlation analysis was then used to identify significant interactions between nasal cell transcriptomes and the nasal microbiome and how these interactions were altered in animals with CR. Notably, we observed significant downregulation of multiple genes associated with ciliary function in dogs with CR, suggesting a previously undetected role for ciliary dysfunction in this syndrome. We also found significant upregulation of immune genes related to the TNF-α and interferon pathways. The nasal microbiome was also significantly altered in CR dogs, with overrepresentation of several potential pathobionts. Interactome analysis revealed significant correlations between bacteria in the genus *Porphyromonas* and the upregulated host inflammatory responses in dogs with CR, as well as defective ciliary function which was correlated with *Streptococcus* abundance. These findings provide new insights into host–microbe interactions in a canine model of CR and indicate the presence of potentially causal relationships between nasal pathobionts and the development of nasal inflammation and ciliary dysfunction.

## Introduction

Chronic rhinitis (CR) is a common condition in dogs associated with persistent (weeks to months) nasal discharge ranging from serous to mucopurulent (with or without sneezing) that is often refractory to treatment with antibiotics, antihistamines, or oral steroids. Previous reports indicate that CR may account for up to 24% of all cases of chronic nasal disease in dogs (1–3). The diagnosis of CR in dogs is typically that of exclusion, after other causes for nasal signs including mycotic disease (aspergillosis), neoplasia, foreign body, oronasal anatomical abnormalities (cleft palate, nasopharyngeal polyps, stenosis) and periodontal disease have been ruled out. The histopathology of nasal biopsy tissue often reveals a non-specific lymphoplasmacytic inflammation that does not correlate with either disease severity or response to therapy. The disease in dogs resembles non-allergic rhinitis (NAR) in humans, and specifically idiopathic NAR, with respect to persistent clinical signs without an obvious cause, lack of an obvious allergic response by biopsy or allergy testing, and absence of obvious infections (4–6).

Given the lack of correlation between histopathology findings and treatment responses in dogs, the pathogenesis of CR in dogs remains largely unknown (7) despite two experimental studies suggesting a link to persistent allergy as a possible cause of CR (8, 9). Chronic rhinitis is an inflammatory disorder, based on the typical lymphocytic immune infiltrates observed on nasal biopsies, but the nature of the immune responses or the initiating or perpetuating causes are unknown. Clinically, an allergic basis for CR in dogs seems unlikely given the lack of response to treatment with glucocorticoids, antihistamines, NSAIDs, or cyclosporine (10–12). Upper digestive tract abnormalities may occasionally be detected in a small subset of dogs with CR, postulating a possible association between gastroesophageal reflux and/or lymphoplasmacytic gastrointestinal inflammation (13). Management of CR remains challenging, and the condition is commonly a source of frustration for patients, pet owners, and clinicians. An improved understanding of the pathogenesis of CR is therefore essential to identify better treatments and management strategies, and to help develop a possible canine CR model for study of human idiopathic rhinitis.

Ciliary dyskinesia is a rare but important cause of repeated upper and lower airway infections in humans and dogs (14, 15). The function of cilia has not been widely investigated previously in studies of allergy related CR in humans, and there are few reports of ciliary dyskinesia in dogs (16, 17). However, one common finding in dogs with ciliary dyskinesia is recurrent nasal infections (18). Next generation sequencing technologies now provide unbiased tools to investigate host transcriptomes and microbiome compositions, without the previous reagent limitations posed by conventional immunohistochemical and cytokine analysis approaches in dogs. For example, a previous investigation of the nasal mucosal transcriptome in dogs with CR utilized an earlier generation of RNA transcriptome technology (microarray) and generated important new information, but did not provide the depth of sequence coverage given by current Illumina-based RNA sequencing technologies, such as employed in the current study (19).

Recent studies in humans with chronic allergy-associated CR have characterized the local transcriptome of the nares (20, 21), and it is now evident that such approaches can provide a more comprehensive examination of the molecular mechanisms associated with nasal inflammation in humans or dogs (22). Host and microbe interactions are an important component of the nasal ecosystem, as there is evidence in humans that disruption of the normal nasal microbiome may contribute to the initiation or perpetuation of chronic rhinitis (23). Previous studies of the normal canine nasal microbiome (24–27), reported that the dominant phyla included *Proteobacteria*, *Firmicutes* and *Bacteroidetes*. In dogs with fungal rhinitis, a dysbiotic microbiome was described which included decreased *Morexella* spp. abundance, increased *Pasteurellaceae* spp. abundance, and increased *Lactobacillaceae* abundance (28). Evidence from prior microbiome studies in humans with chronic rhinosinusitis suggests that the abundance of certain microbes may be related to the persistence of nasal inflammation (29). However, to our knowledge there has only been one report in human nasal disease where the host immune transcriptome was correlated directly with the microbiome of the nasal mucosa (30).

The goals of the present study were to characterize and compare the nasal mucosal transcriptomes and microbiomes of healthy dogs with those of dogs with CR, to provide a better understanding of the pathogenesis of CR in dogs. We hypothesized that both the nasal transcriptomes and the microbiomes in dogs with CR would be significantly different from those of healthy dogs, and that disease specific correlations between specific bacterial populations and host immune response genes would be identified in dogs with CR, and would differ significantly from those observed in healthy animals.

## Materials and methods

### Study population

In this prospective case-control study, dogs with CR (*n* = 6) and healthy control dogs (*n* = 6) presented to Colorado State University Veterinary Teaching Hospital (CSU-VTH) for medical workup (CR) or routine dentistry (healthy controls) were screened for study enrolment. The protocols for this study were approved by the CSU Institutional Animal Care and Use Committee and the CSU-VTH Clinical Review Board (protocol #2858). Inclusion criteria for CR dogs included at least 1-month history of unilateral or bilateral nasal discharge, congestion, and/or sneezing. Histologic documentation of lymphoplasmacytic rhinitis with or without concurrent neutrophilic or eosinophilic rhinitis based on nasal mucosal biopsies, without evidence of neoplasia, fungal infection or other identified causes of CR, based on CT and rhinoscopy, was also required for study entry.

Exclusion criteria included any animals receiving immune suppressive medications, including glucocorticoids, leflunomide, mycophenolate, azathioprine, or cytotoxic chemotherapy in less than a month prior to the enrolment. Animals with previously diagnosed endocrine diseases (Cushing’s, diabetes mellitus) that might alter nasal immunity were also excluded. Dogs with pronounced epistaxis were also excluded. A diagnosis of other diseases that may cause the nasal signs (neoplasia, aspergillosis, foreign body, oronasal fistula, nasopharyngeal stenosis, chronic regurgitation) were also criteria for exclusion.

A control group of *n* = 6 dogs was selected from animals presented to the CSU-VTH for routine dental cleaning, with no reported nasal signs or other respiratory signs, gastrointestinal signs or dental diseases that may affect the nasal cavity (e.g., oronasal fistula) were included as the healthy control group. All animals enrolled had no antibiotic usage for 30 days prior to swab collection.

### Sample collection and processing

Nasal swabs were collected under general anesthesia by gently swabbing the distal third of the nasal cavity using 6-inch PurFlock Ultra, sterile flocked collection devices (Puritan Medical Products, Guilford, ME). Swabs (2 swabs obtained per each nostril) were placed in 15 mL conical tubes (Corning Inc. Corning, NY) containing RNA later (Thermo Fisher Scientific, Waltham, MA) and stored at 4C prior to RNA and DNA extraction. To extract RNA from nasal cells, swabs were vortexed for 1 min at high speed to dislodge cells, PBS was then added to RNA later at a ratio of 1:5. Cells were pelleted, then processed for RNA extraction using Qiagen RNeasy micro kit (Qiagen, Hilden, Germany) following manufactures instructions or DNA extraction as described below.

### RNA sequencing

RNA concentrations were verified on Nanodrop 1000 Spectrophotometer (Thermo Fisher Scientific), and then sent to Novogene Corp. Inc. (Sacramento, CA) for RNA sequencing. RNA quality was determined using an Agilent 2100 Bioanalyzer system to generate RIN numbers (RNA integrity number), which ranged from 6.9 to 10 for all RNA samples submitted. At Novogene Corp., mRNA was purified using poly-T oligo-attached magnetic beads. After fragmentation, the first strand cDNA was synthesized using random hexamer primers followed by the second strand cDNA synthesis. The library was completed following end repair, A-tailing, adapter ligation, size selection, amplification, and purification. Quantified libraries were pooled and sequenced on an Illumina NovaSeq 6000 (Illumina, San Diego, CA). 150 bp paired end reads were generated, and files were delivered as de-multiplexed fastq files.

Sequence data were analyzed on Partek Flow software, version 10.0 (Partek Inc. Chesterfield, MO). Raw data were filtered by removing reads containing adapters and reads containing *N* > 10% and for Phred scores >30. Filtered reads were aligned with STAR 2.7.3a, using CanFam3.1 genome assembly. Aligned reads were annotated and counted using HT-seq (31) with Ensembl 107, and differentially expressed genes were identified using DEseq2 (32). (Differential gene expression analysis based on negative binomial distribution). Biological interpretations included gene ontology and gene set enrichment analysis (GSEA).^1^ Gene sets Hallmarks v2022.1, biocarta v2022.1, KEGG v2022.1, Gene Ontology go.bp v2022.1, and ImmuneSigDB v2022.1 were used for comparisons. Significant pathways were filtered using false discovery rate (FDR) *q*-value of≤0.25 and NOM *p*-value ≤0.05. Single cell gene expression data from the Human Protein Atlas^2^ was used to identify olfactory and epithelial cell specific gene sets that were not expressed by immune cells.

Previously published single cell sequencing data from human nasal epithelium (33) was used to identify unique genes in nasal cell types (secretory cells, goblet cells, club cells, ionocytes, ciliary cells and rapidly dividing cells RDC) that were not found in previously published RNAseq data fromcanine peripheral blood mononuclear cells (34).

### Microbial 16S sequencing

Additional swabs were collected for microbial analysis. Swabs were cut and immersed in extraction tubes following Qiagen DNeasy PowerSoil Pro Kit instructions (Qiagen, Hilden, Germany). Microbial DNA was frozen at −80°C and sent to Anschutz Center for Microbiome Excellence (ACME), at University of Colorado Anschutz Medical Campus, Aurora, CO for microbial sequencing. The library was prepared according to Earth Microbiome project protocol,^3^ with 35 PCR cycles, using 515F and 806R primers. Samples were run on MiniSeq cartridges on Illumina Miseq sequencing instruments. Microbial sequence analyses were performed with QIIME2 (35). Microbial community similarity was displayed with principal coordinate analysis (PCoA) plots. Alpha diversity was determined using Shannon, Faith, and pielou indices. Beta diversity using weighted and unweighted UniFrac, as well as Bray Curtis. Alpha diversity indices were compared using a paired *t*-test, and beta diversity metrics were compared with PERMANOVA. Analysis of composition of microbiomes (ANCOM) was employed to determine the sequence variants that differed significantly between treatment groups (36). In addition, Linear discriminant analysis Effect Size (LEfSE) was also used to calculate the taxa that best discriminated between rhinitis or healthy group^4^ (37). Because of the low overall bacterial abundance in the swab samples, additional PCR cycles were needed to reach the required number of reads for each sample. Microbial features were filtered for a minimum frequency of 50 were removed and features not present in >2 samples were also removed, resulting in a total of 106 total features. Median frequency for *n* = 12 samples was 14,455.5 and total frequency was 228,553.

### Assignment of cell identity using Cibersort

Single cell RNA sequence data from *n* = 17 dogs (38) was used to generate a signature matrix file for Cibersort analysis of immune cells in the nasal swabs (39). Using the data from 74,067 immune cells in blood that were obtained previously from scRNAseq analysis from 17 dogs, 41 transcriptionally distinct leukocyte populations were identified. For analysis of leukocyte populations in dog nasal samples, each of the 41 cell populations were downsampled to obtain equal representation across each cell type, then normalized count data for each population was summed up to obtain a cell type by gene matrix. After collapsing count data by cell type, the dataset was filtered to only include the most highly variable features that define each cell population. Filtering was completed using the output of the FindAllMarkers function, with features being selected if they had an average log2 fold change (relative to all other cells) greater than 0.85 and a percent expression greater than 75% within the cell population. After initial filtering, any overlapping features were then removed from the dataset. This data set was then uploaded to^5^ along with the median ratio normalized counts from the current study to determine immune cell type enrichment (similar datasets were not available for epithelial cell populations in dogs).

### Interactome analysis of transcriptome and microbiome data

For this analysis, significant differentially expressed genes (DEGs) were extrapolated from the RNA sequencing data. These median ratio normalized reads from individual samples were then correlated to the percent relative abundance of 160 unique bacterial taxon found in *n* = 12 samples (healthy and rhinitis) using rcorr (Rstudio) (40). *p* values for significance and *r* values for correlation were generated for each gene to taxon pair. Protein coding genes with correlations *p*-values ≤0.05 were then entered into string protein database^6^ (41) for categorizing the protein sets.

### Data availability

The RNA sequencing data including fastq files and read counts presented in this manuscript have been deposited in NCBI’s Gene Expression Omnibus and are accessible through GEO Series accession number GSE255529. All other data is available from the corresponding author on reasonable request.

## Results

### Study populations and relevance to CR

To address the question of how the nasal transcriptome and microbiomes may be altered in dogs with CR, we designed a prospective study to sequence deep nasal swab samples from *n* = 6 healthy dogs and *n* = 6 dogs with CR of at least 1 month duration. The demographic data of the CR dogs and heathy dogs are listed in Tables 1, 2. This study was approximately matched for age and gender, though the healthy dogs were overall younger than CR dogs. Within the CR group, the severity of clinical signs ranged from mild to erosive and more severe CR (Table 1).

**Table 1:** Demographic data of dogs of chronic rhinitis.

Dog	Breed	Age	Sex	Weight (kg)	Duration of clinical signs (months)	Histopathology	Antibiotics	Anti-inflammatory drugs
CR1	Mixed Breed	13 Y	MN	14.2	3	Plasmacytic and eosinophilic rhinitis, moderate with edema	Doxycycline	Carprofen
CR2	Miniature Dachshund	9 Y	FS	5.5	14	Mild lymphoplasmacytic rhinitis with few neutrophils	Doxycycline	Prednisolone
CR3	Mastiff	7 Y	MN	97.6	25	Mild to moderate, multifocal chronic lymphoplasmacytic rhinitis	Clindamycin	Carprofen
CR4	German Shepherd Dog	10.5 Y	FS	31.2	12	Lymphoplasmacytic rhinitis, erosive, chronic, marked with subcucosal edema	Ciprofloxacin, amoxicillin /clavulanic acid	None
CR5	Mixed Breed	9 Y	FS	19.0	2	Moderate, chronic-active, lymphoplasmacytic and neutrophilic rhinitis	Doxycycline, clindamycin	Prednisone
CR6	Golden Retriever	12 Y	MN	32.1	1	Chronic-active lymphoplasmacytic rhinitis, severe with mucosal erosion and loss	None	Meloxicam

Y, years; MI, male intact; MN, male neutered; FS, female spayed.

**Table 2:** Demographic data of healthy control dogs.

Dog	Breed	Age	Sex	Weight (kg)
HC1	Mixed breed	5 Y	FS	16.4
HC2	Cavalier King Charles Spaniel	5 Y	MN	7.8
HC3	Labrador Retriever	9 Y	FS	30.4
HC4	Australian Shepherd	5 Y	MI	18.1
HC5	Belgian Tervuren	7 Y	MN	26.8
HC6	Australian Heeler	1 Y	FS	14.5

Y, years; MI, male intact; MN, male neutered; FS, female spayed.

### Nasal transcriptome analysis reveals marked differences in expression of immune and epithelial genes in dogs with CR, along with a reduced abundance of eosinophils

**Figure 1:**
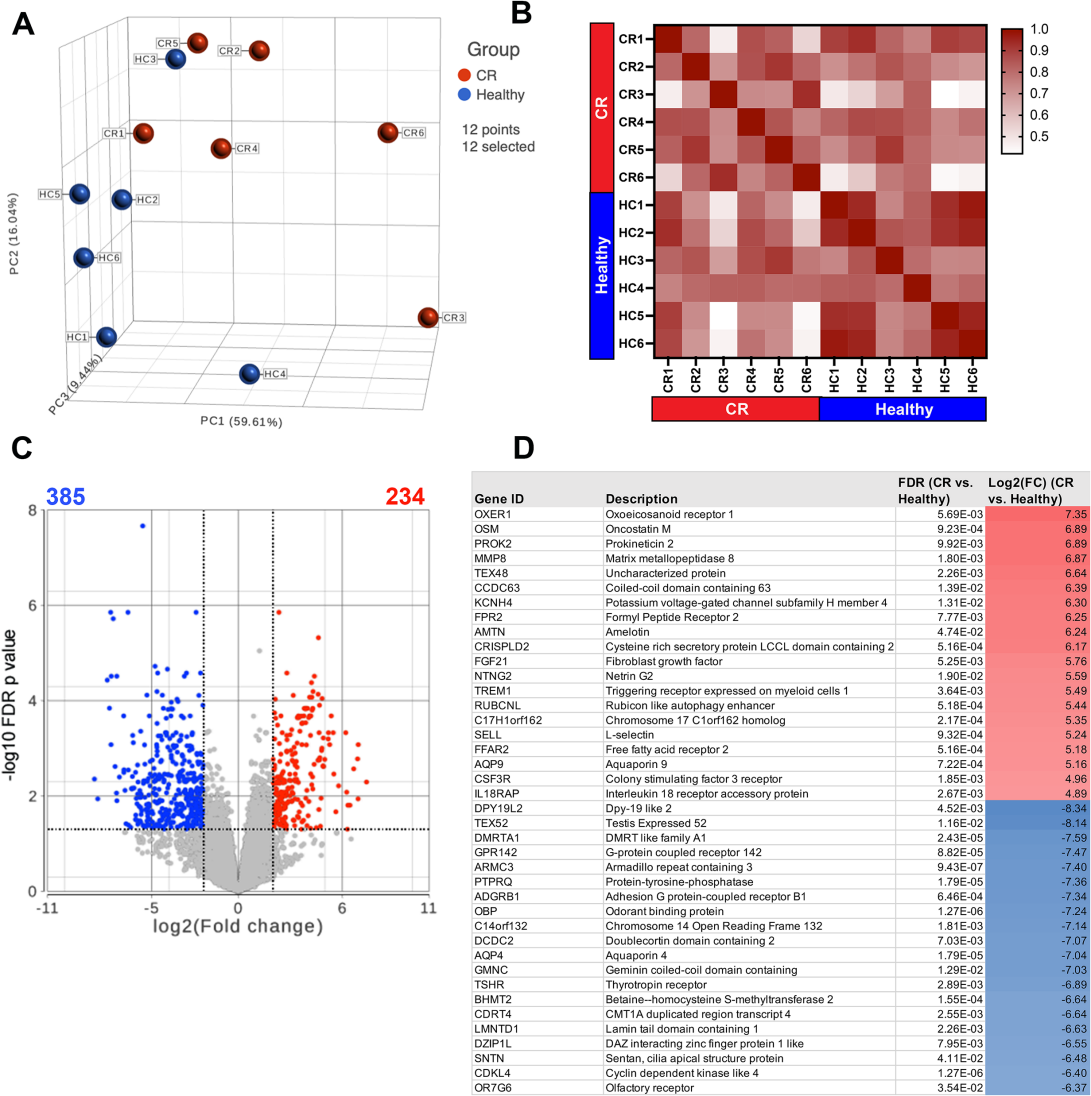
RNA sequencing of nasal swab cells. Transcriptome analysis was performed on cells swabbed from 6 confirmed rhinitis and 6 healthy dogs. **(A)** PCA plot of rhinitis samples (red) compared to healthy (blue) with 17,975 detectable genes. **(B)** Sample similarity index with values from 0.42 to 1 represented in red gradient. Sample names and disease state labels included in color. **(C)** Volcano plot of significant genes from DEseq analysis of *n* = 6 rhinitis vs. *n* = 6 healthy samples. Red dots show significant genes defined by fold change >2Log2, FDR ≤0.05. Blue dots show significantly downregulated genes defined by fold change <−2Log2, FDR ≤00.05. **(D)** List of top 20 significantly upregulated or downregulated genes (rhinitis vs. healthy), shown with gene description, FDR adjusted *p*-values, Log2 fold change.

**Figure 2:**
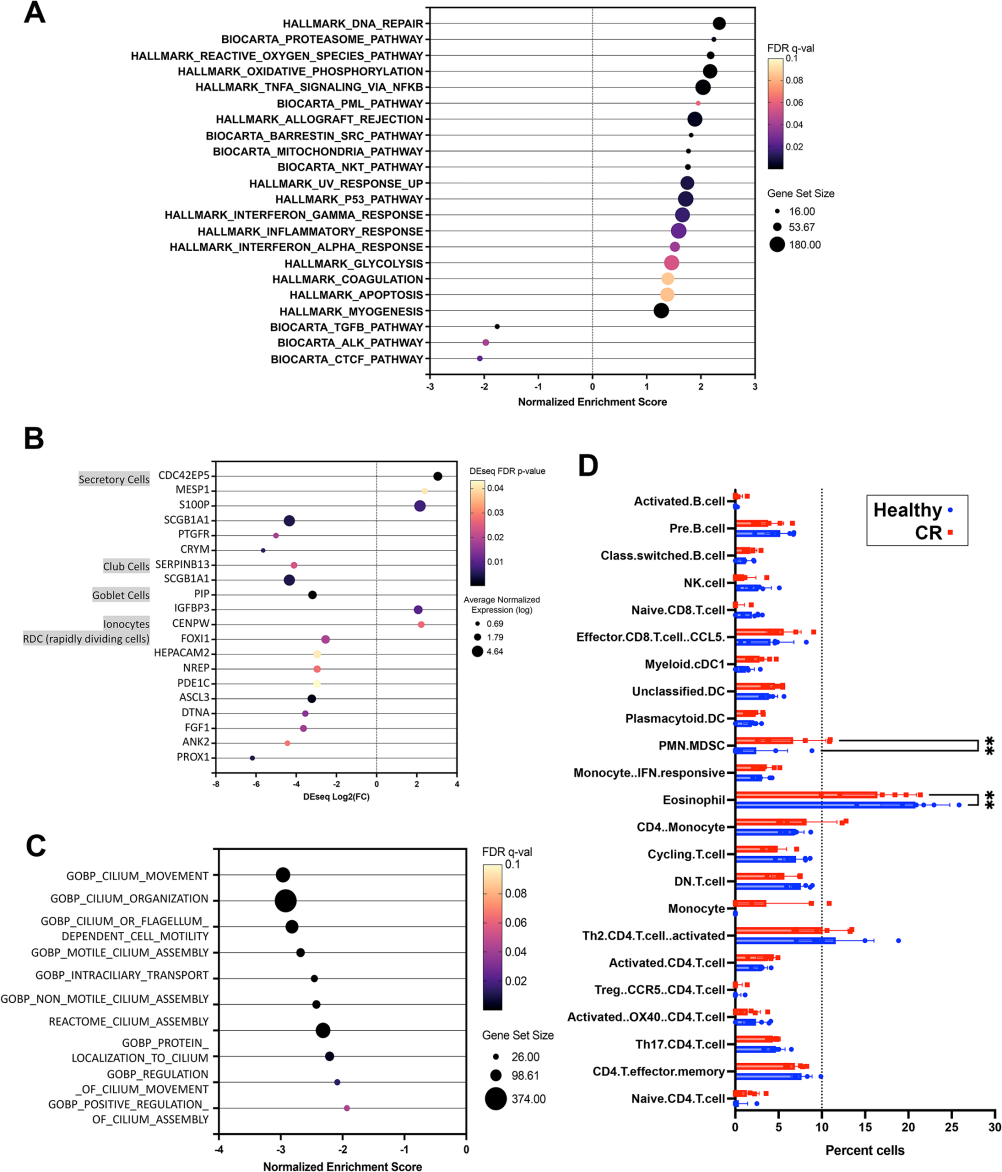
Pathway and cell type analysis for nasal transcriptome. **(A)** GSEA analysis results using Hallmarks and Biocarta pathways. Significant pathways organized by NES (normalized enrichment score) shown on *x*-axis from negative 3 to 3. FDR adjusted *p*-value shown in color scale, size of circles showing total genes represented. **(B)** Nasal epithelium specific genes with significantly differential expression in CR vs. HC comparison. Dot size shows normalized expression, *x*-axis shows Deseq fold change results (CR vs. HC). Color scale for FDR adjusted *p*-value (all <0.05). **(C)** Significant GSEA pathway associated with cilium, all downregulated in CR, normalized enrichment score (NES) shown on *x*-axis with total genes in pathway represented by dot size. **(D)** Cibersort was used to define cell types expressed in canine rhinitis or healthy samples represented in red and blue respectively. Normalized transcriptome count for 17,975 protein coding genes were used for analysis. Significance between disease vs. healthy groups was calculated using two way ANOVA with Bonferroni multiple comparisons test. *X*-axis shows cell types defined by single cell transcriptome data as described in methods, with dotted line on 10% total cell population. **(C)** Dot plot of nasal epithelium specific genes. Size shows noramalized expression value (average *n* = 11), FDR *p*-value shown in color (all <0.05).

The first question addressed in this study was whether the transcriptomes of nasal mucosal cells, including epithelial cells, immune cells, and stromal cells, were significantly different in CR dogs compared to healthy. A total of 17,975 protein coding genes were identified in the 12 analyzed samples. The samples from the *n* = 6 CR dogs showed distinct clustering compared to the *n* = 6 healthy dogs (Figure 1A) with a high degree of transcriptome heterogeneity present in the CR samples. By contrast (Figure 1B) the heathy nasal transcriptomes displayed a relatively high level of within-sample homogeneity. Next, differential gene expression (DEseq) analysis was done comparing CR transcriptomes to healthy transcriptomes (based on analysis of 14,504 protein coding genes with known annotation). We found 234 significantly upregulated genes with a fold-change (FC) ≥ 2 Log_2_ and FDR adjusted *p*-value of ≤ 0.05. The analysis also revealed 385 genes whose expression was significantly downregulated, with FC ≤ −2 Log_2_ and FDR ≤ 0.05 (Figure 1C). The most upregulated genes in the CR samples included genes encoding OXER1, OSM, PROK2, MMP8 and TEX48, while the genes with the most downregulated expression included OR7G6, CDKL4, SNTN, DZIP1L and LMNTD1 (Figure 1D). Pathway analysis of the differentially expressed immune-related genes (DEG) revealed upregulation of inflammatory pathways such as the TNF-α and IFN-γ and IFN-α pathways in the CR animals, along with significant downregulation of cell cycle (CTCF) and activin receptor kinase (ALK) and TGF-β pathways in CR dogs (Figure 2A).

Epithelial cell dysfunction may also be an important contributor to the pathogenesis of CR, so we also analyzed the epithelial cell transcriptomes in study dogs. To conduct this analysis, we interrogated human single cell databases (33) for epithelial cell specific genes, including those associated with mucous and serous glandular cells, salivary duct cells, ciliated cells, squamous epithelial cells. Our analysis also excluded genes expressed by canine leukocytes (34). From this analysis, we found that there were numerous genes (117 in total) associated with ciliated airway cell function whose expression was significantly downregulated in dogs with CR (Supplementary Figures S2A,B). In addition, genes associated with cilia and flagella associated proteins, mucin and olfactory receptors were also downregulated (Supplementary Figure S2C), along with genes expressed by other nasal epithelium specific cell types (Figure 2B). Pathways related to cilium assembly, movement and regulation were also found to be downregulated in samples from dogs with CR (Figure 2C). These findings suggest that ciliary dysfunction and defective clearance of airway mucus and debris may play an important role in CR pathogenesis in dogs. However, the current study was not able to determine whether ciliary dysfunction was a primary or secondary occurrence in dogs with CR.

In addition to bulk transcriptome analysis, a Cibersort matrix generated from canine single cell leukocyte sequencing data (38) was used to deconvolute the bulk RNAseq data from the nasal swabs in the present study into gene sets derived from specific immune cell populations. Using Cibersort analysis, we identified a total of 23 different immune cell populations in the 12 RNAseq samples from healthy dogs and dogs with CR. We found that the percentage of polymorphonuclear-myeloid derived suppressor cells (PMN-MDSC) was significantly higher in nasal swabs from dogs with CR compared to healthy dogs, while unexpectedly the percentage of eosinophils was significantly lower in dogs with CR compared to healthy dogs (Figure 2D).

### Analysis of nasal microbiomes reveals significant differences in dogs with CR compared to healthy dogs

**Figure 3:**
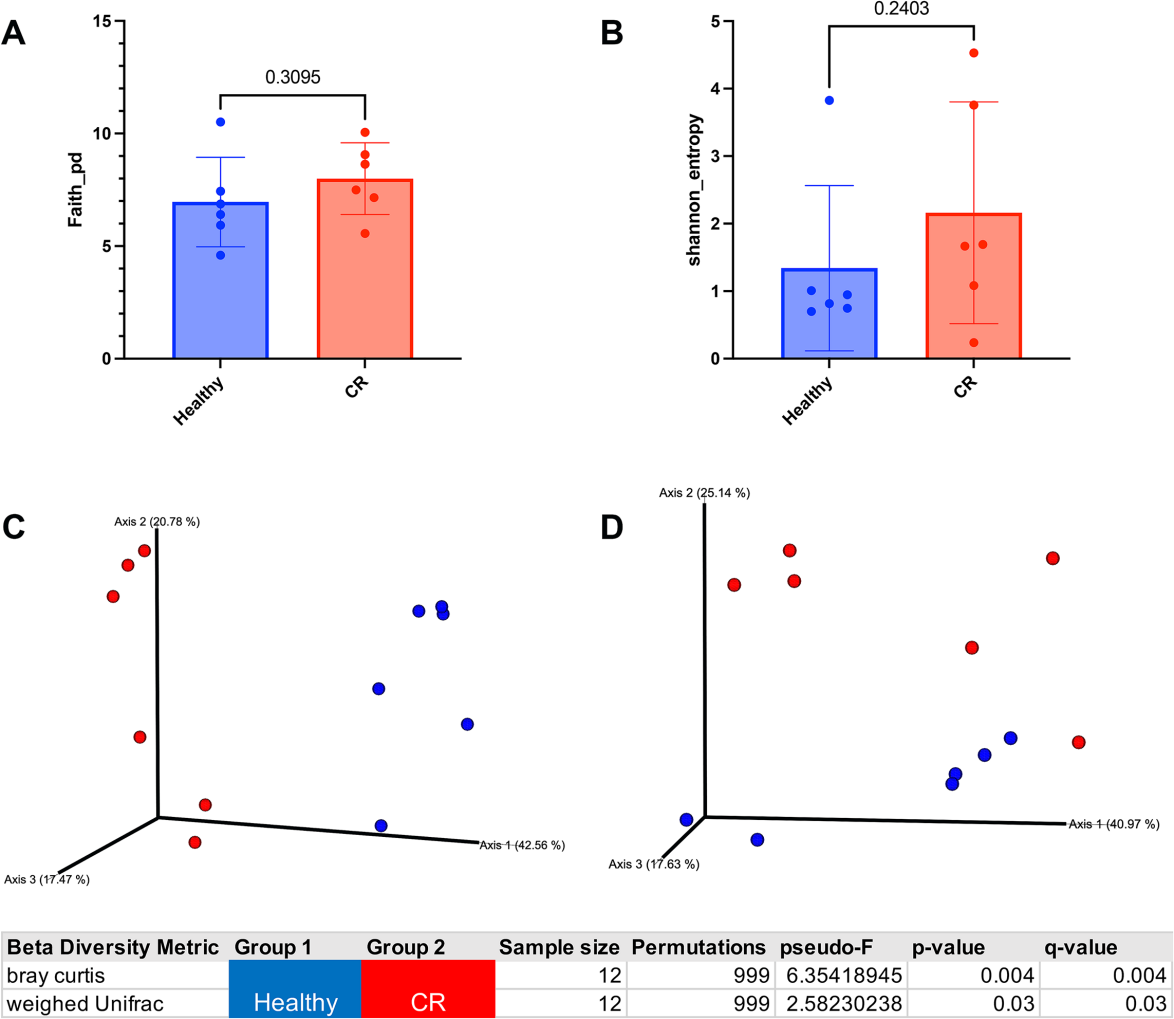
Alpha and beta diversity analysis of healthy and rhinitis microbiome. **(A)** Faith alpha diversity metrics of healthy (blue) compared to rhinitis. Phylogenetic diversity (PD) Statistical differences compared using non-parametric Mann Whitney *t*-test. Exact *p*-value shown in graph. **(B)** Shannon diversity metrics of healthy or rhinitis microbiome. **(C)** 3D Bray Curtis beta dissimilarity pCoA plots generated for beta diversity comparison. Healthy samples labeled in blue and rhinitis in red. Table shows *p*-value for significant differences between healthy and rhinitis samples. **(D)** 3D weighed UniFrac PCoA plots generated for beta diversity comparison. Significant *p*-value shown in bottom table.

Microbial 16S rRNA sequencing revealed 160 unique taxa in the nasal swab samples. To assess the overall diversity in bacterial populations between groups, the alpha and beta diversity were compared between CR and healthy samples, which revealed that there were no significant differences in alpha diversity, either using faith or Shannon metrics (Figures 3A,B). However, beta diversity was significantly different between CR and healthy samples, with distinctly separated clusters of the CR and healthy samples for Bray Curtis beta diversity and for weighed UniFrac distance measurement (Figures 3C,D). These analyses therefore indicated the presence of a dysbiotic bacterial flora in the nose of dogs with CR, which has been reported previously (24).

**Figure 4:**
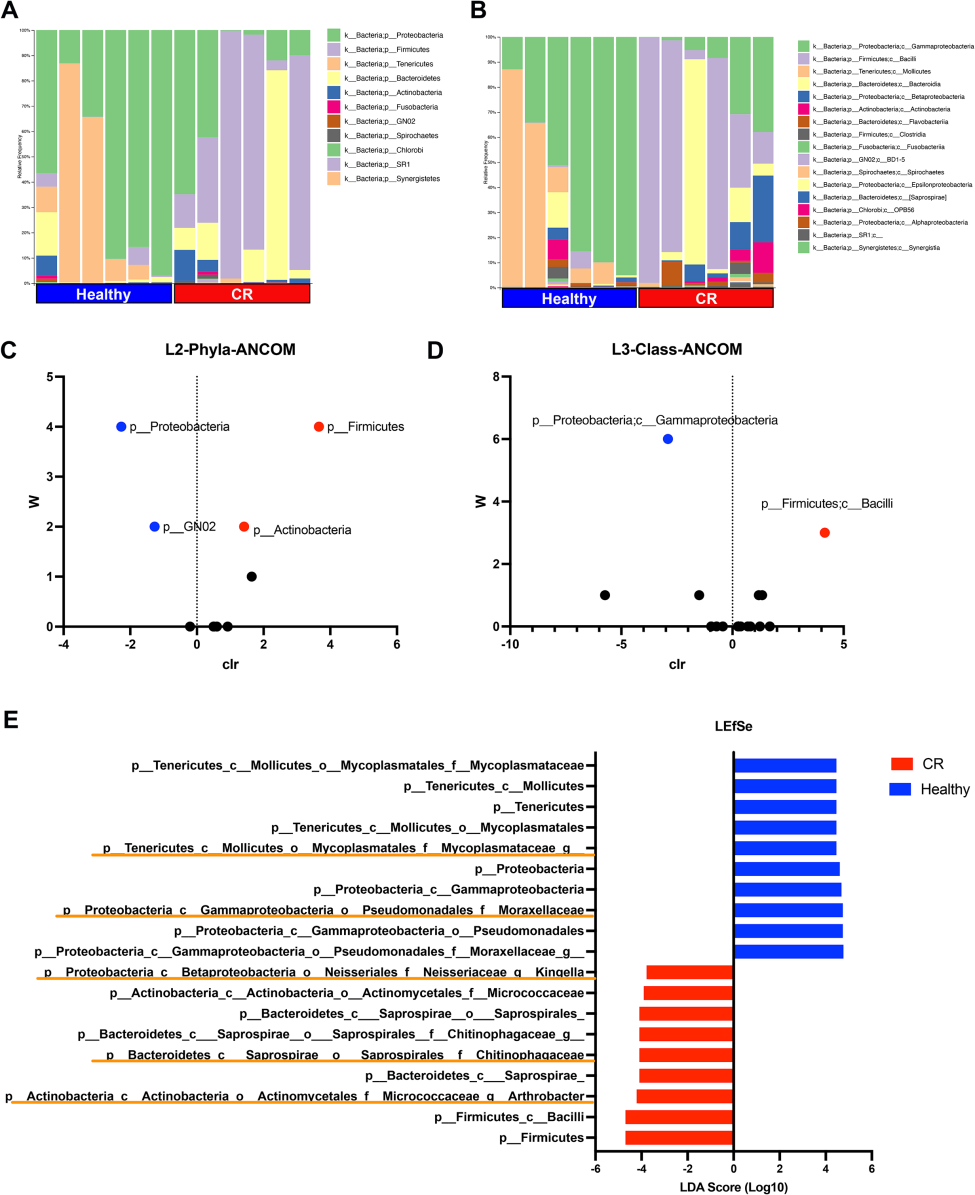
Microbial taxonomy distribution for rhinitis and healthy nasal microbiome. **(A)** Phylum level taxonomy represented in stacked bar plots. Healthy and rhinitis samples grouped together. Colors show relative percent abundance of phyla, labels shown on right. **(B)** Class level taxonomy in stacked bar graphs with labels on right. **(C)** ANCOM analysis volcano plot comparing healthy to rhinitis, relative abundance on phyla level. *Y*-axis *W* value represents the number of times of the null-hypothesis is rejected. *X*-axis value represents the centered log ratio (clr) transformed mean difference in relative abundance. Significantly upregulated phylum in health samples labeled in red, and significantly downregulated phylum in healthy samples in blue. **(D)** ANCOM differential abundance volcano plot on the class level. **(E)** LefSEe results showing LDA scoring of significantly different taxon abundance between heathy (blue) and rhinitis (red). Orange highlights show unique genus level differences not found in ANCOM.

Of the 11 phyla identified in the nose of dogs with CR and in healthy dogs, the most abundant phylum was *Proteobacteria* in the healthy dogs and *Firmicutes* in dogs with CR (Figure 4A). Four phyla were found to be significantly different in terms of relative abundances between dogs with CR and healthy dogs, as assessed by ANCOM analysis (Figure 4C). These included *Proteobacteria* and *GN02* which had a significantly higher abundance in dogs with CR, whereas *Firmicutes* and *Actinobacteria* were significantly less abundant in dogs with CR compared to healthy dogs (Figure 4C).

Sixteen class level taxon were identified, with the most abundant class being *Gammaproteobacteria* in healthy dogs and *Bacillus* in dogs with CR (Figure 4B). Significant differences were found by ANCOM analysis between CR and healthy dogs for both of these most abundant bacterial classes (Figure 4D). Linear discriminant analysis Effect Size (LEfSe) was also used to interrogate the differences in bacterial populations, and this analysis revealed additional family and genus level differences which significantly contributed to the altered mucosal bacterial populations present in CR samples. For example, *Arthrobacter* and *Kingella* family *Chitinophagaceae* had higher relative abundance in the CR group, while the family *Mycoplasmataceae* had a greater relative abundance in the healthy control group (Figure 4E).

### Interactome analysis reveals significant interactions between bacterial populations and host immune response and ciliary pathways in the dog nasal mucosa

Finally, we used the nasal transcriptome and microbiome data to develop interaction mapping to better understand potential interactions between host nasal responses and microbial populations. First, the DEGs (see Figure 1) were selected from the transcriptome data, and the normalized expression values for 619 differentially expressed genes were then correlated to the relative abundance percentage of 160 bacterial taxa. This analysis revealed that 502 host genes in the nose exhibited a significant correlation (*p* ≤ 0.05) with at least a single bacterial taxon.

**Figure 5:**
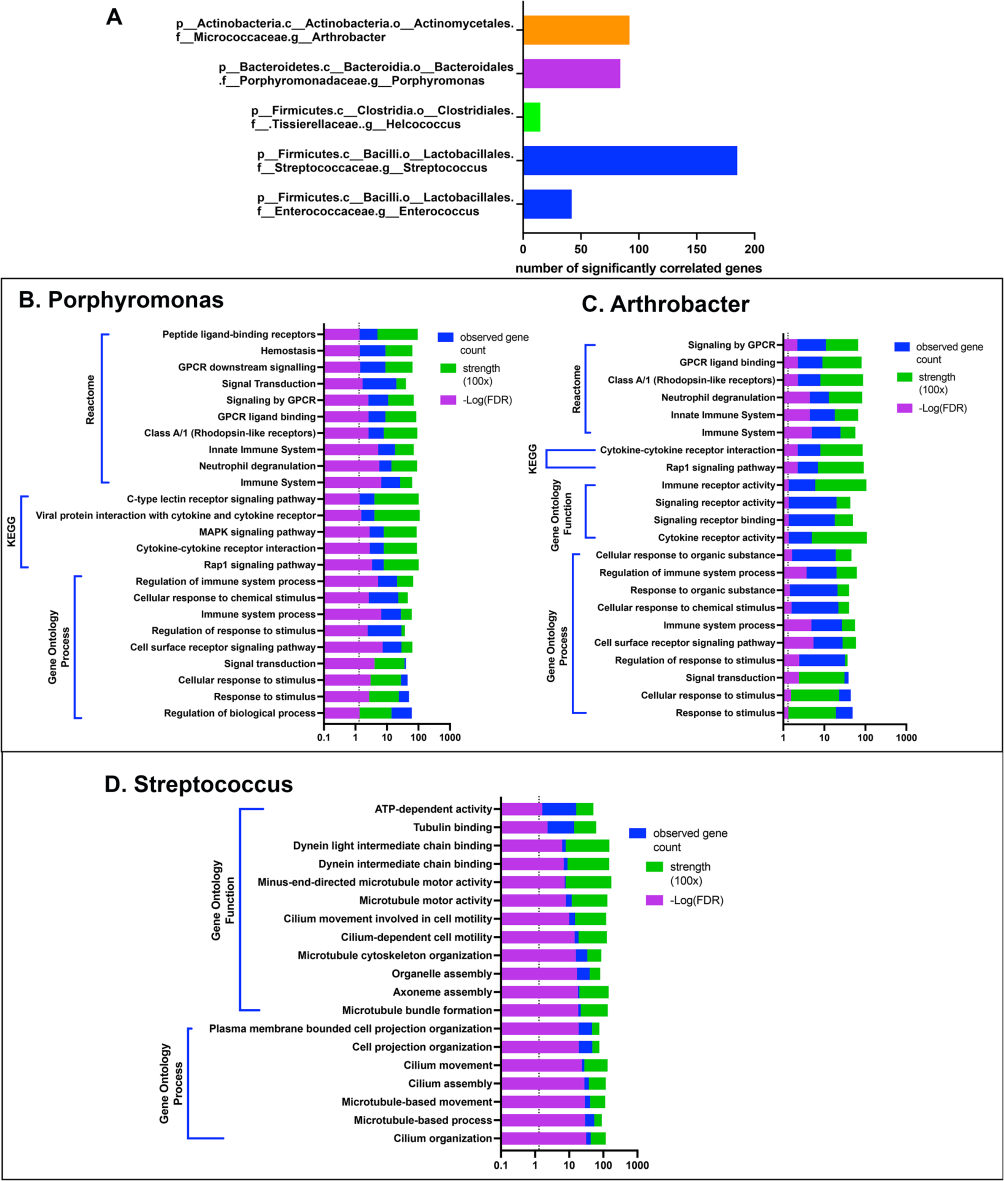
Gene based interactome of canine rhinitis. Interaction between transcriptome and microbiome (interactome). Using significant DEGs (637) genes in total, correlation was performed between taxon abundance and normalized gene count. **(A)** Shows 5 taxon at the genus level with over 15 significant gene correlations. *X*-axis scale bar with total significantly correlated genes. **(B–D)** String.db was used to find gene set enrichments for correlated genes for each 5 genera shown. **(B)** Shows categorization results for 84 genes with positive correlation to *Porphyromonas*, including GO, KEGG and reactome. Legend on top right, significant FDR *p*-value in reverse log scale (purple), total gene count matched to pathways in blue, and signal strength measurement in green. **(C)** Bar graph of categorization results of 92 genes with significant positive correlations to genus arthrobacter. **(D)** Bar graph of categorization results from 185 genes with significant correlation to the abundance of genus streptococcus.

Five bacterial genera, including *Enterococcus*, *Streptococcus*, *Helcococcus*, *Porphyromonas*, and *Arthrobacter*, exhibited significant correlations with 15 differentially expressed genes (Figure 5A). These potentially interacting genes were then entered into the String protein database to find meaningful connections or interactions. Protein network association showed enrichment for host gene sets amongst 3 of the 5 bacterial genera. Interestingly, the gene sets that correlated with *Porphyromonas* and *Arthrobacter* mapped to multiple immune pathways (Figures 5B,C). In contrast, the gene sets correlated with *Streptococcus* involved primarily motility and cytoskeletal structure related genes, including many of the ciliary pathways found to be downregulated in CR dogs (Figure 5D). Thus, our interactome analysis revealed several important host and bacterial interactions, involving both immune and nasal epithelial cell responses and particular populations of bacteria, including some (e.g., Porphyromonas) previously identified as pathobionts.

## Discussion

In the present study, we analysed the nasal mucosal transcriptomes and microbiomes of dogs with CR help elucidate the pathogenesis of the syndrome and better define host–microbe interactions. One important and unexpected finding was evidence of significantly downregulated expression of multiple genes related to ciliary and flagellar function in dogs with CR (Figure 1), suggesting that ciliary dysfunction might be an important overall contributor to the pathogenesis of the syndrome in dogs. We also found that multiple inflammatory pathways were upregulated in dogs with CR, including TNF-α and interferon signalling pathways, involving both type I and II interferons.

To our knowledge, the present study is also the first in which the interaction of the host immune response and a mucosal microbiome population has been investigated in dogs. The interactome analysis revealed significant interactions between nasal cells and the pathobiont *Porphyromonas* in dogs with CR, suggesting a possible causal role for this organism, which has been reported previously for dental disease in dogs and humans (42, 43). Interactome analysis also identified a significant interaction between the *Streptococcus* sp. and ciliary dysfunction, again indicating a strong association and possible causal role in CR. These findings illustrate the value of applying next gen sequencing tools to help elucidate microbe and host response interactions at sites of mucosal inflammation, including the nose, the pharynx, the upper airways, and the female reproductive tract. Moreover, our findings suggest the possibility of new, creative therapeutic interventions for managing CR in dogs, including the use of targeted antimicrobial therapy and locally applied immunotherapy (44, 45).

Among the more surprising findings was the apparent broad downregulation of expression of genes related to ciliary and flagellar function in dogs with CR. These findings suggest significant dysfunction in mucus clearance mechanisms in dogs with CR (46, 47). Humans and dogs with inherited ciliary dyskinesia experience repeated bouts of upper and lower respiratory tract infection that are difficult to manage clinically (16, 18, 46). In the present study, it was not possible to determine whether ciliary dysfunction was a primary cause of CR in dogs, or whether dysfunction was a secondary pathology resulting from sustained nasal inflammation and transient or permanent loss of cilia. For example, it has been reported that inflammation can induce ciliary dysfunction, as can toxins produced by certain bacteria such as *Pseudomonas* sp. (46, 48). Regardless, impaired ciliary clearance of mucous and pathogens may be an integral part of the CR pathogenesis.

The immune transcriptome analysis revealed that OXER1 was the single most upregulated immune gene in the nasal mucosa of dogs with CR. This gene has been implicated in inflammation (49), playing an important role in the migration of neutrophils and monocytes. Other genes that were significantly upregulated in the nose of dogs with CR including OSM and MMP8, and IL-18RAP, each of which has been associated with mucosal inflammation (50, 51). Notably, OSM and OSMR have been reported to be a key immune pathways dysregulated in inflammatory bowel disease in humans (52). In addition, upregulated expression of the genes CSF3R and SELL in dogs with CR, which are associated with neutrophil migration, have been shown to mediate inflammatory responses in nasal epithelial cells in humans (53). Such findings may suggest a possible therapeutic role for leukocyte function antigen-1 (LFA-1) inhibitors such as fuzapladib in management of CR, despite the fact that corticosteroid therapy is largely ineffective for treatment.

Surprisingly, in the current study there was no transcriptome evidence to suggest the presence of allergic responses in dogs with CR, as determined by either DEG analysis or immune pathway analysis. Instead, the predominant pathways upregulated in dogs with CR were IFN-γ, IFN-α, TNF-α and ROS pathways, all of which are more consistent with a Th1 immune activation pathway. Moreover, based on Cibersort analysis, eosinophils were significantly depleted in the nose of dogs with CR. These findings are also consistent with the lack of clinical benefit provided by corticosteroids and antihistamines management of CR in dogs, in agreement with what has been reported for idiopathic CR in humans (54). In addition, downregulation of expression of ciliary and olfactory genes suggests a mechanical aspect of CR pathogenesis, mediated at least in part by epithelial surface dysfunction. Further exploration of the transcriptomic patterns within a larger patient population of dogs with CR may be required for further subclassification of canine CR, and to confirm the findings reported in this study.

The altered microbiome of the nasal cavity in dogs with CR has been reported previously, and one study reported a decreased relative abundance of *Proteobacteria*, driven largely by decreased abundance of the *Moraxella* family (19), which agrees with the findings of our study. Our phylum abundance findings were also consistent with those of Tress et al. (24), wherein it was reported that there was decreased *Proteobacteria* and increased *Bacteroides* abundance in CR samples in dogs. Interestingly, *S. aureus* and *S. pneumoniae*, which are known pathobionts for nasal disease processes in humans (55) were not identified as significant contributors to the CR microbiome in the current study.

The interactome analysis done in this study provided critical context to understanding the potential impact of dysbiosis on the pathogenesis of CR dogs. By itself, microbiome analysis provides relatively little insight into disease mechanisms, whereas when the host response is factored into the microbiome analysis, new and potentially important connections can be identified. For example, several recent studies have explored the upper airway microbiome and transcriptome interactome in children with asthma (56–60). In dogs, *P. gingivalis* has been identified as a canine pathobiont in the context of periodontal disease (61). Moreover, *Porphyromonas* is increasingly being recognised as a constituent of the core lung microbiome in humans (62). Previous studies have shown an association with heme and ion transport related genes in *P. cangingivalis* and *P. canoris*, with the former being the most abundant bacteria in the oral cavity of dogs (61). In the current study, of the 81 host genes with a positive corelation to *Porphyromonas*, the 3 genes with the highest correlation (CSF-1, CCL3, FPR2) were all genes involved in host inflammatory responses.

Despite the observed significant interaction between *Porphyromonas* and host immune responses in dogs with CR, the overall abundance *of Porphyromonas* was not significantly different between healthy and CR animals. This finding suggests that it may not be the relative abundance of *Porphyromonas* organisms in the nasal cavity that is the primary driver of immune responses, but rather the relative pathogenicity of *Porphyromonas* strains may evolve over time of nasal colonization, particularly under the influence of antibiotic pressure (63) or the presence of deleterious strains or sub-strains (64). However, we do note that two dogs with CR that had higher abundance of *Porphyromonas* each had increased neutrophilic infiltration. These early findings suggest therefore that *Porphyromonas* may function as an opportunistic pathobiont in the nose of dogs, in addition to its known role in the pathogenesis of dental disease.

In a previous study in human asthma patients, a significant increase in *Streptococcus* abundance was noted nasal samples, and downregulation of ciliary function genes in children with asthma compared to healthy controls (59). Likewise, we observed a significant interaction between *Streptococcus* and ciliary function genes in dogs with CR. We also noted that the presence of *Streptococcus* correlated with downregulation of expression of host genes known to be related to ciliary cell structure and motility (dynein, tubulin, ATP cilium). These findings taken together suggest that both *Porphyromonas* and *Streptococcus* are associated with ciliary dysfunction in dogs with CR and suggest that indeed these organisms may play a causal role in CR in dogs.

The major limitation of this study was the small sample size for each population, which may lead to spurious disease and microbe associations. The small sample size may also lead to an underestimation of the full complexity of immune and microbe changes in dogs with CR. Nonetheless, even with the small sample set we were able to identify significant and consistent differences in both host responses and microbiome populations in dogs with CR. In addition, the median age of the healthy control group was significantly lower compared to the CR dogs (5 years vs. 9.75 years). Although there is precedence for age associated microbial changes in human CR patients (65), the sample size in the dog CR group was too small for comparisons between older and younger CR dogs.

In summary, this study provides new insights into the host and microbe interactions that occur in the nose of dogs with CR. Particularly noteworthy was the widespread evidence of ciliary dysfunction in dogs with CR, along with the lack of evidence of allergic inflammation. Thus, these new findings suggest a rethinking of the role of allergy in CR and dogs and indicate the need to refocus our understanding of CR pathogenesis on the potential role of pathobionts in the disease. The interactome analysis provided early evidence of important connections between potential pathobionts in the nasal cavity and sustained nasal inflammation. If confirmed, such findings could pave the way for targeted antimicrobial therapy, including intranasal antibiotic delivery, or the use of pathobiont targeted vaccines. Alternative approaches could also employ locally delivered innate immune activating immunotherapy to strengthen or alter the host immune responses to nasal pathobionts (45, 66).

## Data availability statement

The data presented in the study are deposited in the GEO repository, accession number GSE255529.

## Ethics statement

The animal studies were approved by CSU Institutional Animal Care and Use Committee and the CSU-VTH Clinical Review Board. The studies were conducted in accordance with the local legislation and institutional requirements. Written informed consent was obtained from the owners for the participation of their animals in this study.

## Author contributions

ZW: Conceptualization, Data curation, Investigation, Methodology, Project administration, Resources, Validation, Visualization, Writing – original draft, Writing – review & editing. LC: Conceptualization, Data curation, Formal analysis, Funding acquisition, Investigation, Methodology, Project administration, Resources, Software, Supervision, Validation, Visualization, Writing – original draft, Writing – review & editing. SDa: Data curation, Formal analysis, Methodology, Writing – original draft, Writing – review & editing. RI: Investigation, Methodology, Writing – original draft. AM: Writing – original draft, Writing – review & editing. SDo: Conceptualization, Data curation, Formal analysis, Funding acquisition, Investigation, Methodology, Project administration, Resources, Software, Supervision, Validation, Visualization, Writing – original draft, Writing – review & editing.

## Footnotes

^1^ https://www.gsea-msigdb.org/gsea/index.jsp

^2^ https://www.proteinatlas.org

^3^ https://earthmicrobiome.org/protocols-and-standards/16s/

^4^ https://huttenhower.sph.harvard.edu/lefse/

^5^ https://cibersortx.stanford.edu

^6^ https://string-db.org

## Supplementary material

SUPPLEMENTARY FIGURE S1 Heat map of TH1 and Th2 genes from CR and healthy samples. Gene expression levels from high (red) to low (blue).

SUPPLEMENTARY FIGURE S2 Heat maps of ciliary cell genes. **(A,B)** Ciliary cell genes unique to the nasal epithelium that were significantly downregulated in dogs with CR. **(C)** Additional genes with key word associated with “cilia, olfactory, mucin, or keratinocyte.”
