# Supplementary figures and images for: Host–microbe interactions in the nasal cavity of dogs with chronic idiopathic rhinitis
Source: Front Vet Sci. 2024 Aug 12;11:1385471. doi: 10.3389/fvets.2024.1385471 (PMC11345268; doi:10.3389/fvets.2024.1385471)

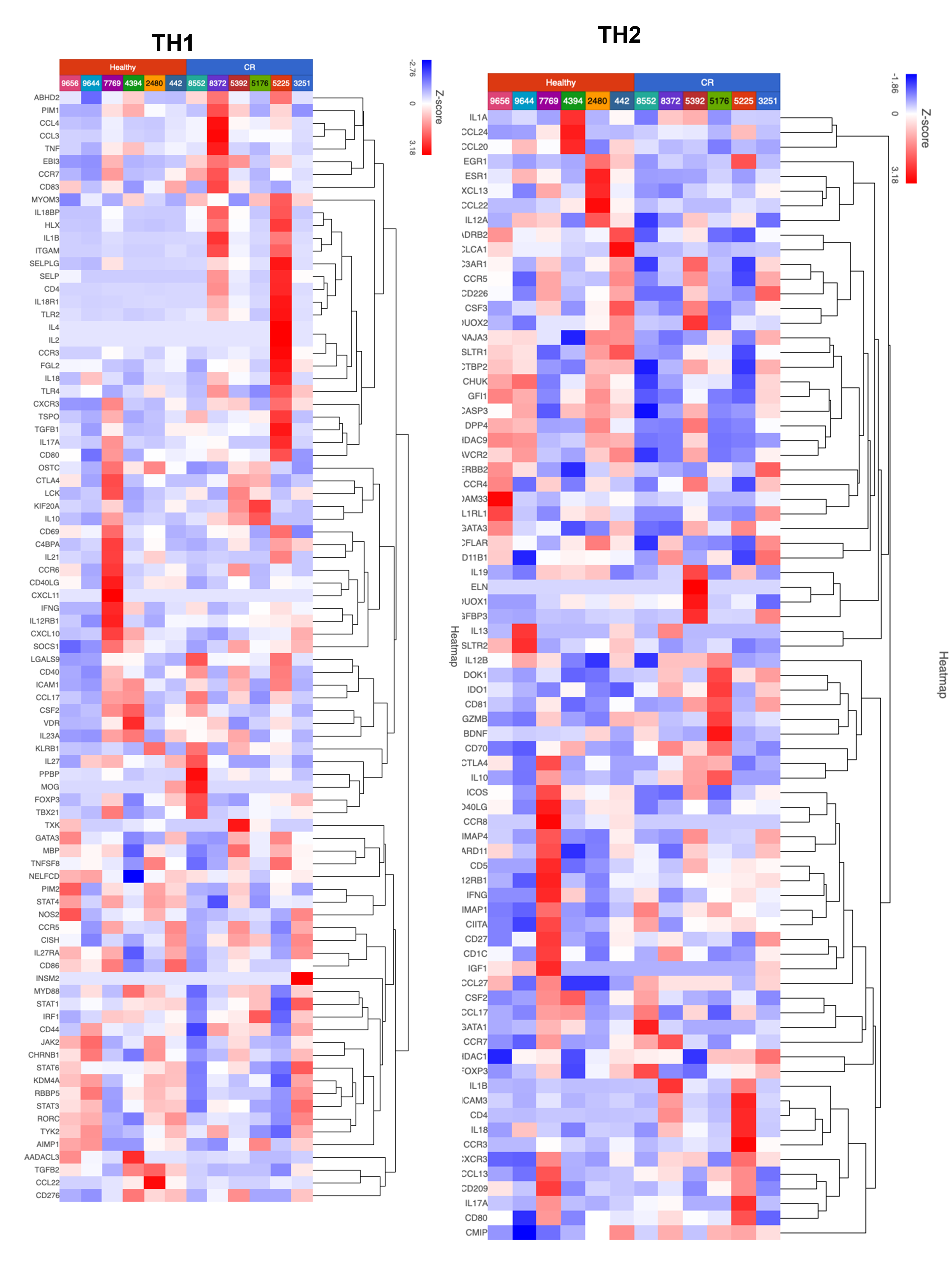

Supplement: SUPPLEMENTARY FIGURE S1 — Heat map of TH1 and Th2 genes from CR and healthy samples. Gene expression levels from high (red) to low (blue). [file Image_1.TIF]

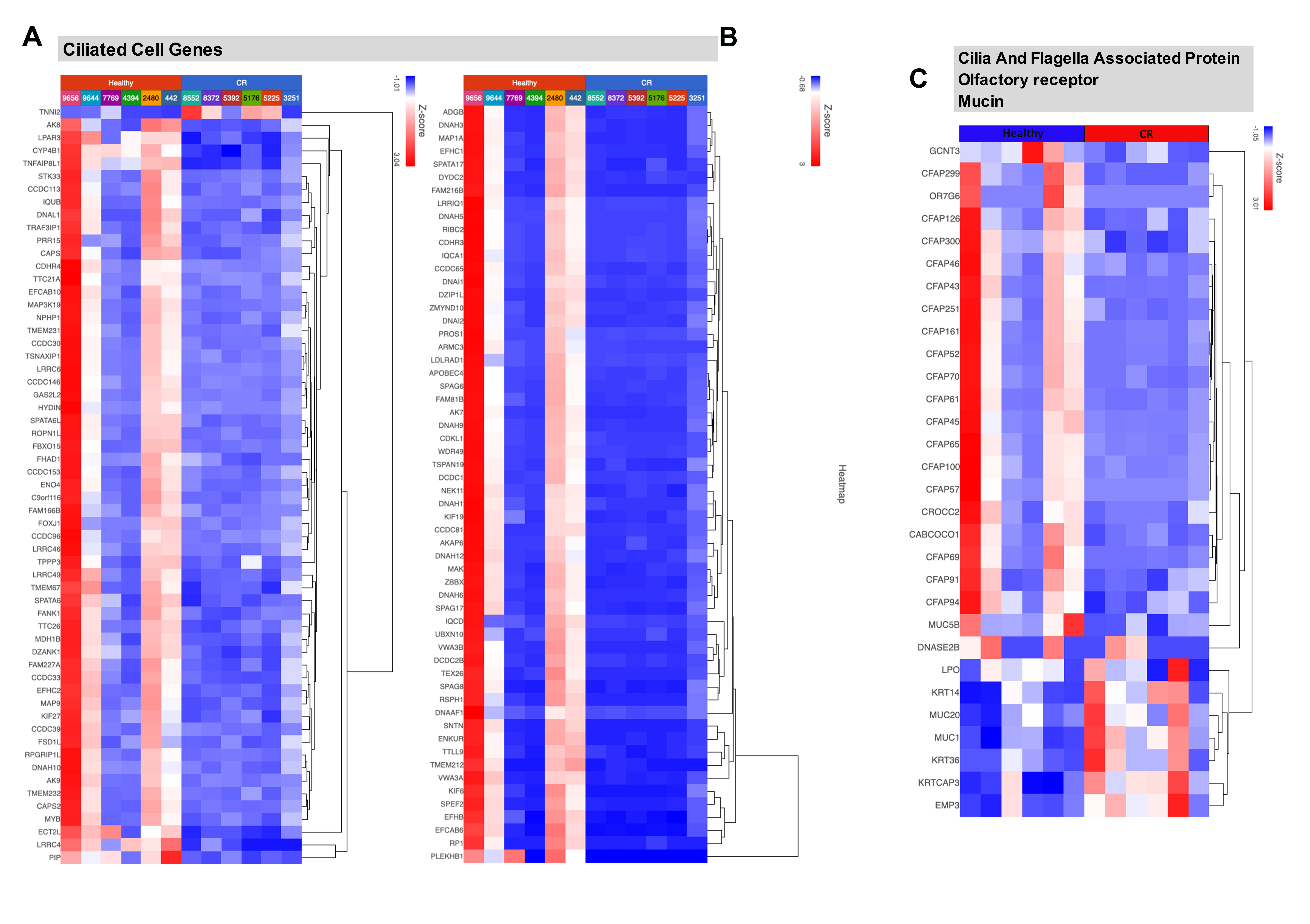

Supplement: SUPPLEMENTARY FIGURE S2 — Heat maps of ciliary cell genes. (A,B) Ciliary cell genes unique to the nasal epithelium that were significantly downregulated in dogs with CR. (C) Additional genes with key word associated with “cilia, olfactory, mucin, or keratinocyte.” [file Image_2.TIF]
